# Maternal Exposure Results in Long-Term Deoxynivalenol Persistence in Piglets’ Plasma and Modulates the Immune System

**DOI:** 10.3390/toxins12100615

**Published:** 2020-09-25

**Authors:** Hana Štěpánová, Karolina Hlavová, Kamil Šťastný, Eduard Gopfert, Lenka Levá, Martin Faldyna

**Affiliations:** Veterinary Research Institute, 62100 Brno, Czech Republic; stepanova@vri.cz (H.Š.); hlavova@vri.cz (K.H.); stastny@vri.cz (K.Š.); gopfert@vri.cz (E.G.); leva@vri.cz (L.L.)

**Keywords:** deoxynivalenol, pig, intrauterine exposure, immune system, T lymphocytes, cytokines

## Abstract

Deoxynivalenol (DON)-contaminated feed represents a serious problem for pigs due to their high sensitivity to its toxicological effects. The aim of the present study was to evaluate the impact of intrauterine DON exposure on the immune system of piglets. Pure DON was intravenously administered to sows at the end of gestation (during the last 2–3 days of gestation, one dose of 300 µg per day). The plasma concentration of DON was analyzed using liquid chromatography combined with high-resolution Orbitrap-based mass spectrometry (LC–MS/MS (HR)) and selected immune parameters were monitored six times in piglets from birth to 18 weeks. DON was found in the plasma of 90% of newborn piglets at a mean concentration of 6.28 ng/mL and subsequently, at one, three, and seven weeks after birth with decreasing concentrations. Trace amounts were still present in the plasma 14 weeks after birth. Flow cytometry revealed a significant impact of DON on T lymphocyte subpopulations during the early postnatal period. Lower percentages of regulatory T cells, T helper lymphocytes, and their double positive CD4+CD8+ subset were followed by increased percentages of cytotoxic T lymphocytes and γδ T cells. The capacity to produce pro-inflammatory cytokines was also significantly lower after intrauterine DON exposure. In conclusion, this study revealed a long-term persistence of DON in the plasma of the piglets as a consequence of short-term intrauterine exposure, leading to altered immune parameters.

## 1. Introduction

Deoxynivalenol (DON), also known as vomitoxin, is a secondary metabolite of molds. *Fusarium* species are the main source of this mycotoxin, which preferentially contaminates wheat, maize, and barley. DON is very stable and persists on the grain for a long time. Animal feed made from contaminated grain poses a serious threat to the health of the animal, as well as having an economic impact. Recognizing this, the European Union set guidance values for DON in feed in the Commission Recommendation No. 2006/576/EC [1]. Animal species show different sensitivity to DON, from relatively tolerant species such as poultry and ruminants, to pigs being the most sensitive farm animals [2,3]. Additionally, its metabolism differs depending on the animal species. DON is metabolized via several biotransformation pathways, including conjugation to glucuronic acid (GlcAc), sulfate, or sulfonate. While glucuronidation prevails as the major phase II metabolic pathway in humans, pigs, and ruminants, sulfation dominates in poultry [4]. By in vitro incubation of liver microsomes from various species, the formation of three glucuronides has been demonstrated: DON-5-GlcAc (humans), DON-3-GlcAc (bovine, rats, fish, porcine, humans, and chickens), and DON-7-GlcAc (bovine, rats, and fish) [5]. Later, the major novel compound iso–DON-3-GlcAc was detected in rat, mouse, and pig urine, which had most likely previously been misidentified as DON-7-GlcAc [6]. DON can also be metabolized by gut microbes. The most prominent microbial metabolite of DON is deepoxy-DON (DOM-1) [7]. Microbial de-epoxidation is especially important in ruminants, but is also found in pigs and poultry [8].

The toxicological effect of DON is multifactorial, with exposure in pigs potentially causing vomiting, reduced feed intake, and gastroenteritis, resulting in low body weight gain [9,10,11]. Data from studies carried out in mice models show that DON affects the gastrointestinal hormones related to appetite [12] and increases the plasma levels of anorexic hormones, including cholecystokinin (CCK) [13,14]. Furthermore, the immunostimulatory or immunosuppressive effects have been shown to be a result of DON exposure, depending on the dose [15,16,17]. At the cellular level, the immunomodulatory effects of DON are believed to be mediated through the ribotoxic shock response, primarily via the activation of kinases associated with ribosomes, a primary cellular target of DON [18].

Significant economic losses in pork production also result from the influence of DON on reproductive performance [19,20,21] when the developing fetus is exposed due to pregnant sows ingesting a toxin-contaminated diet [22,23]. However, a subsequent detailed study showed that no pathomorphologically or immunohistochemically detectable alterations occur in fetal organs after intrauterine transfer of DON [24]. Likewise, other studies showed that the exposure to DON-contaminated feed has either no or only a limited impact on pigs. The unaltered performance and gut physiology of weaned piglets exposed to DON were described by Pasternak et al. [25]. A low DON (maximum 840 µg/kg of feed) dose has been shown not affect the hematological, biochemical, and immune parameters in weaned piglets [26] and also no effect on the health and production of pregnant sows has been observed [27].

The aim of our recent study was to bring a new insight into intrauterine DON exposure in piglets. DON was intravenously administered to sows at the end of gestation, and the presence of DON in the plasma of the piglets was evaluated from birth to slaughter. DON plasma concentration was correlated with selected immune parameters.

## 2. Results

### 2.1. Deoxynivalenol Concentration in the Plasma of the Piglets after Intrauterine Exposure

**Figure 1:**
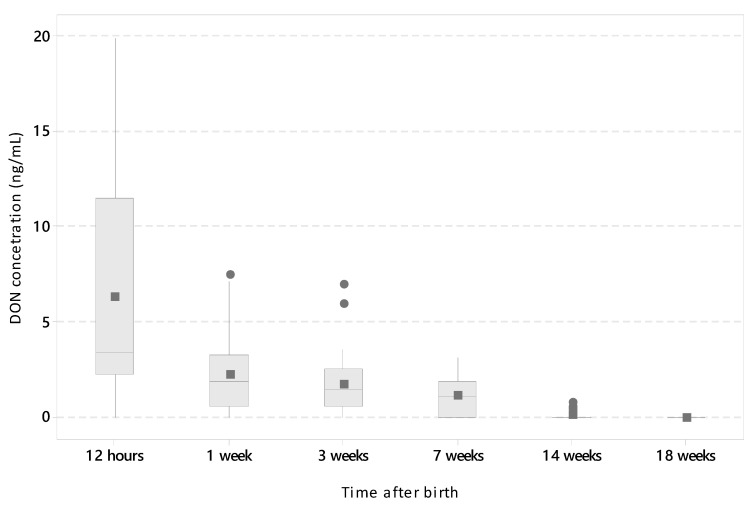
Deoxynivalenol (DON) concentrations in the plasma of the piglets after intrauterine exposure monitored from birth to 18 weeks of life. Data acquired by LC–MS/MS (HR) are expressed as nanograms per milliliter (*y*-axis; DON concentration). The number of piglets in each age category (*x*-axis; time after birth) was as follows: 12 h, *n* = 44; 1 week (1 w), *n* = 39; 3 weeks (3 w), *n* = 38; 7 weeks (7 w), *n* = 32; 14 weeks (14 w), *n* = 32; and 18 weeks 18 (18 w), *n* = 32. Boxplot: median (line), mean (square), Quartiles 1 and 3 (box), minimum and maximum (whiskers), and outliers (individual points).

Piglets were exposed to DON at the end of gestation (2–3 days before delivery on a daily basis) as a result of intravenous DON administration to sows. The DON concentration in the plasma of the piglets was measured by LC–MS/MS (HR) at several time points after delivery (12 h and 1, 3, 7, 14, and 18 weeks). Forty-four piglets from four injected sows and 40 piglets from control sows were used in this part of the study. The piglets from the control litters were negative for DON (less than the limit of detection (LOD); LOD = 0.24 ng/mL for plasma) at all six tested time points. In the experimental group, the highest levels (mean and standard deviation (SD) = 6.28 ± 5.76 ng/mL) were seen soon after delivery (12 h) and 90.9% of the piglets were positive for DON. High individual variability was typical at this point. DON was still present in the serum of the piglets during the first week (2.24 ± 1.97 ng/mL) with 83.3% being DON-positive. In the third and seventh weeks, 81.1% (1.74 ± 1.56 ng/mL) and 68.8% (1.13 ± 1.05 ng/mL) of piglets were positive, respectively. Even at 14 weeks, DON was still present in the plasma in trace amounts (0.08 ± 0.21 ng/mL) in 14.8% of the piglets. All pigs were found DON-negative (<LOD; LOD = 0.24 ng/mL) from 18 weeks after birth (Figure 1). Correlation age versus DON concentration was significant (*p* < 0.001; r = −0.705; Spearman’s rank correlation coefficient). To conclude, the short-term intrauterine exposure caused a long-term persistence of DON in the blood of the piglets.

### 2.2. Deoxynivalenol Concentration in the Plasma of the Piglets after Intraperitoneal Exposure at 35 Days of Life

**Figure 2:**
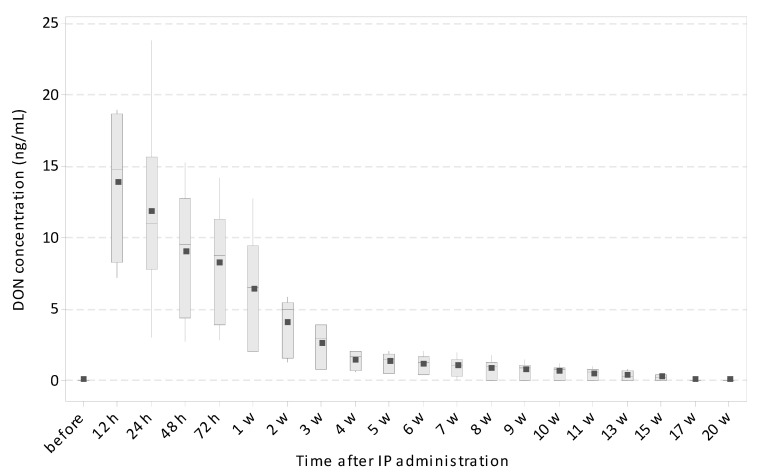
Deoxynivalenol concentration in the plasma of the piglets after intraperitoneal (IP) administration at 35 days of life, monitored from 12 h to 20 weeks after the administration. Data acquired by LC–MS/MS (HR) are expressed as nanograms per milliliter (*y*-axis; DON concentration). The data from all time points (*x*-axis; time after IP administration) were obtained from six animals. Boxplot: median (line), mean (square), Quartiles 1 and 3 (box), and minimum and maximum (whiskers).

To determine if DON exposure around the weaning period caused long-term persistence in the blood of the piglets similarly to intrauterine exposure, a second experiment was carried out. DON was applied intraperitoneally (IP) to six piglets at 35 days old and the amount present in their plasma was measured by LC–MS/MS (HR) at several time points after administration (12, 24, 48, and 72 h and 1, 2, 3, 4, 5, 6, 7, 8, 9, 10, 11, 12, 15, 17, and 20 weeks). All piglets included in this second experiment were negative for DON (<LOD; LOD = 0.24 ng/mL for plasma) at the beginning of the experiment. Not surprisingly, the highest DON concentration was detected a few hours after administration (mean ± SD = 13.78 ± 5.08 ng/mL after 12 h, 11.80 ± 6.83 ng/mL after 24 h, 8.95 ± 4.61 ng/mL after 48 h, and 8.16 ± 4.21 ng/mL after 72 h) followed by a decreasing tendency over the following three weeks. The first negative animal occurred at Week 7. The concentration of DON in positive pigs ranged between 0.96 and 0.21 ng/mL during Weeks 7 and 15. All pigs were negative for DON after 17 weeks, i.e., at 22 weeks old (Figure 2). Correlation of age versus DON concentration was significant (*p* < 0.001; r = −0.682; Spearman’s rank correlation coefficient). To conclude, five-week-old piglets exposed to pure DON via IP administration showed a very similar long-term persistence of DON in their blood as piglets after intrauterine DON transfer.

### 2.3. Plasma Enzymes Aspartate Aminotransferase (AST) and Alanine Aminotransferase (ALT) Determination

The possibility of liver damage caused by intrauterine transfer of DON to piglets was investigated through the measurement of the aspartate aminotransferase (AST) and alanine aminotransferase (ALT) enzymes in the plasma. Three-week-old piglets after intrauterine DON exposure were examined. Although a significant difference was found between the DON and the control groups in both ALT and AST (Mann–Whitney test, *p* < 0.05; control *n* = 33, DON *n* = 38), the reference ranges were not exceeded for AST or for ALT. The values (mean ± SD) found were as follows: AST = 0.591 ± 0.159 μkat/L and ALT = 0.504 ± 0.096 μkat/L in the control group; AST = 0.453 ± 0.159 μkat/L and ALT = 0.433 ± 0.089 μkat/L in the DON group.

### 2.4. Hematological Parameters

The hematological parameters were determined in the piglets after intrauterine exposure to DON at three time points: 1, 3, and 18 weeks after birth. Parameters including red blood cells (RBCs), nucleated red blood cells (NRBC), hematocrit (HTK), hemoglobin (Hb), and total white blood cell count (WBC) were measured by an auto-hematology analyzer and differential leukocyte counts were determined through the analysis of blood smears. No statistically significant differences between the DON and control groups (*p* > 0.05) were observed for any of the monitored hematological parameters when the control and DON groups were analyzed by the Mann–Whitney test. This was observed for all monitored time points (Table 1). To conclude, intrauterine exposure to DON did not cause any changes in the hematological parameters.

### 2.5. Total Immunoglobulin Levels in the Plasma of the Piglets

**Figure 3:**
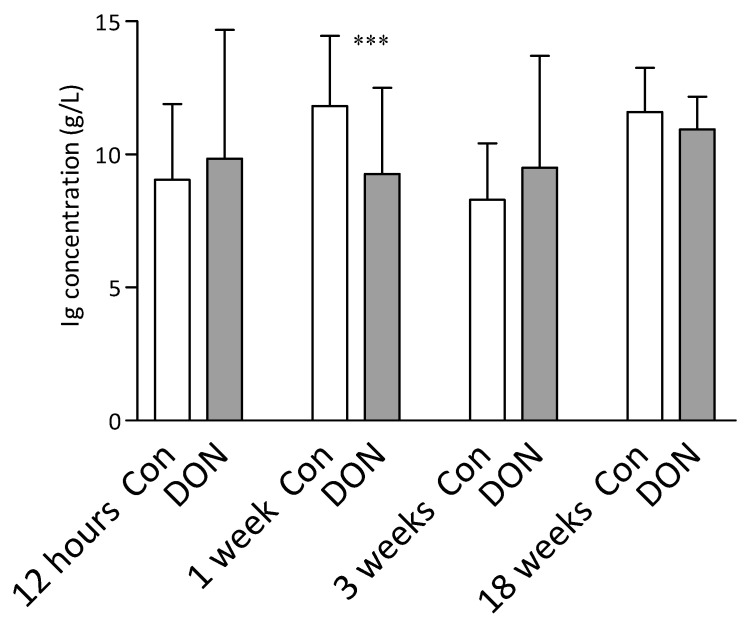
Total plasma immunoglobulin levels detected 12 h and 1, 3, and 18 weeks after intrauterine exposure to DON. The data were obtained from the piglets after intrauterine DON exposure (DON in grey columns) and compared to the control piglets (Con in white columns). Data are expressed as milligrams per milliliter (column) and standard deviation (error bars). The number of piglets in each group was as follows: 12 h control, *n* = 40; 12 h DON, *n* = 44; 1 week control, *n* = 36; 1 week DON, *n* = 39; 3 weeks control, *n* = 33; 3 weeks DON, *n* = 38; 18 weeks control, *n* = 27; 18 weeks DON, *n* = 32. The Mann–Whitney test was used for statistical analysis. Significant differences between the DON and control groups are marked as *** *p* < 0.001.

Total immunoglobulin levels in the plasma of the control and intrauterine DON-exposed piglets were measured 12 h and then 1, 3, and 18 weeks after birth. The only difference in the total immunoglobulin levels between the groups was at one week after birth, where the DON group was significantly lower (*p* < 0.001; Mann–Whitney test) (Figure 3).

### 2.6. Lymphocyte Subpopulations in the Blood of the Piglets after Intrauterine DON Exposure

**Figure 4:**
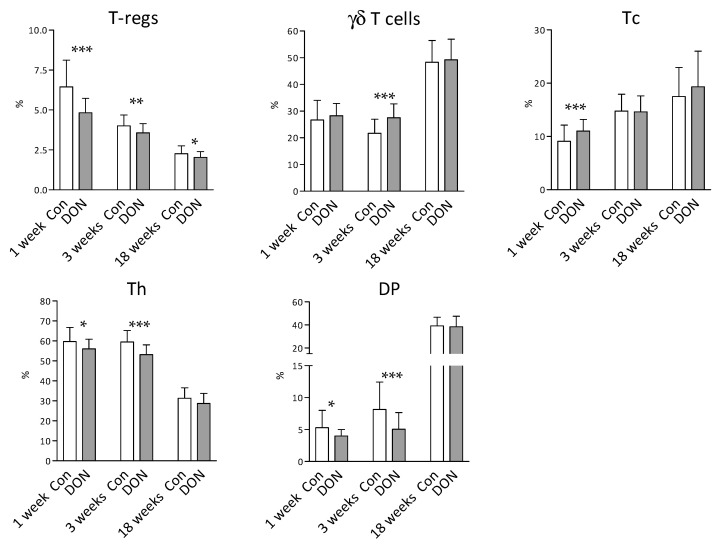
T-lymphocyte subpopulations in the blood of the piglets acquired by flow cytometry. The data were obtained from piglets after intrauterine DON exposure (DON in grey columns) and compared to the control piglets (Con in white columns) at 1, 3, and 18 weeks after birth. Data are expressed as percentage (%) for each lymphocyte population according to the gating strategy (Figure A1). Columns represent the mean and error bars the standard deviation. The number of piglets in each group was as follows: 1 week control, *n* = 36; 1 week DON: *n* = 39; 3 weeks control, *n* = 33; 3 weeks DON, *n* = 38; 18 weeks control, *n* = 27; 18 weeks DON, *n* = 32. The Mann–Whitney test was used for statistical analysis. Significant differences between the DON and control groups are marked as * *p* < 0.05, ** *p* < 0.01, or *** *p* < 0.001. Regulatory T cells (T-regs; CD3+CD4+CD25+FoxP3+), γδ T cells (CD3+γδTCR+CD4-CD8+/-), cytotoxic T cells (Tc; CD3+γδTCR-CD4-CD8+), helper T cells (Th; CD3+γδTCR-CD4+CD8+/-), and double positive CD4+CD8+ cells (DP; CD3+γδTCR-CD4+CD8+).

The effect of intrauterine DON exposure on the T lymphocyte subpopulations in the blood of the piglets was also studied. Flow cytometry revealed many significant differences (*p* < 0.05; Mann–Whitney test) between the piglets from the DON and control groups in the first and third weeks after birth. Significantly lower percentages at both time points (Weeks 1 and 3) were found for regulatory T cells (T-regs; *p* < 0.001 and *p* < 0.01, respectively), CD4+ Th lymphocytes (*p* < 0.05 and *p* < 0.001), and the CD8+ subpopulation of Th cells (DP; *p* < 0.05 and *p* < 0.001) in the piglets from the DON group in comparison to the controls. The percentage of γδ T cells (γδTCR+) cells in the DON group resembled the control group (*p* > 0.05) in the first week but was significantly higher (*p* < 0.001) in the third week after birth. Conversely, the percentages of Tc lymphocytes were significantly higher (*p* < 0.001) in the DON group in the first week and did not differ (*p* > 0.05) in the third week after birth. In contrast to the earlier time points, both groups showed similar proportions of T lymphocyte subpopulations (*p* > 0.05 for the γδTCR+, Tc, Th, and DP populations) 18 weeks after birth (Figure 4). To conclude, intrauterine exposure to DON significantly affects the proportions of T lymphocyte populations in the blood of piglets, and the effect is the most obvious during the early postnatal period.

### 2.7. The Expression of Cytokine mRNA after Non-Specific Stimulation of Blood Leukocytes

**Figure 5:**
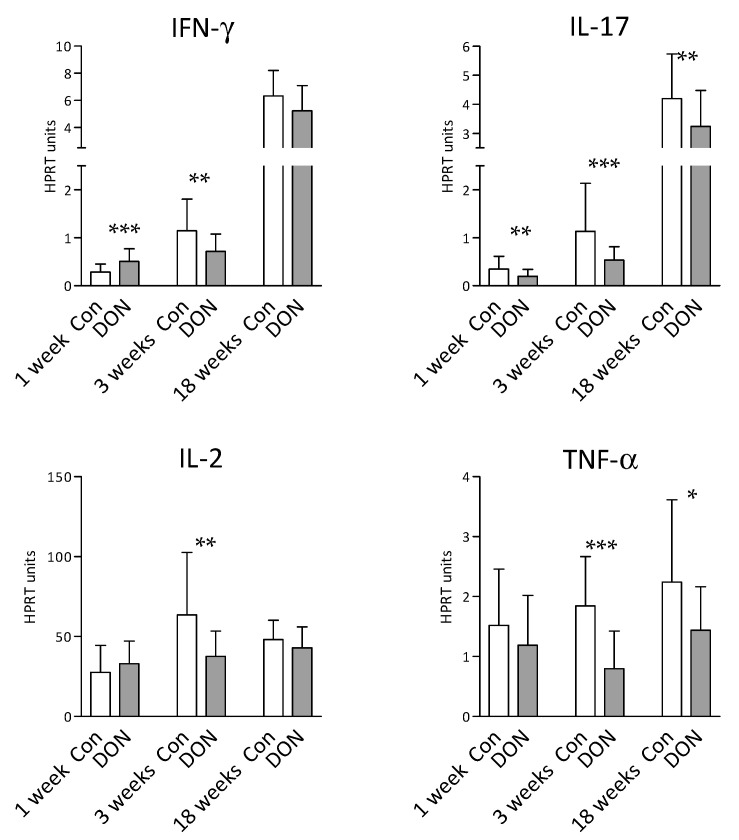
Expression of cytokine (IFN-γ, IL-17, IL-2, and TNF-α) mRNA in the blood leukocytes after non-specific stimulation with PMA and ionomycin. The data were obtained from the piglets after intrauterine DON exposure (DON in grey columns) and compared to the control piglets (Con in white columns) at 1, 3, and 18 weeks after birth. Data acquired by quantitative real-time PCR are expressed as the mean (columns) of multiples of the reference gene expression (HPRT units) and the standard deviation (error bars). The number of piglets in each group was as follows: 1 week control, *n* = 36; 1 week DON, *n* = 39; 3 weeks control, *n* = 33; 3 weeks DON, *n* = 38; 18 weeks control, *n* = 27; 18 weeks DON, *n* = 32. The Mann–Whitney test was used for statistical analysis. Significant differences between the DON and control groups are marked as * *p* < 0.05, ** *p* < 0.01, or *** *p* < 0.001.

Finally, the expression of mRNA for the IFN-γ, IL-17, IL-2, and TNF-α cytokines was determined in the blood leukocytes obtained from the piglets after intrauterine DON exposure. The blood leukocytes were stimulated with PMA and ionomycin, and the cytokines were measured at the mRNA level by real-time quantitative PCR (qRT-PCR). The capacity to produce pro-inflammatory cytokines was determined at three time points: 1, 3, and 18 weeks after birth. A lower IL-17 mRNA level (*p* < 0.01; Mann–Whitney test) was detected in the first week after birth in the DON group, but the differences in IL-2 and TNF-α mRNA levels were non-significant (*p* > 0.05). The expression of the mRNA of all of the monitored cytokines was lower in the DON group in the third week after birth (IFN-γ = *p* < 0.01; IL-17 = *p* < 0.001; IL-2 = *p* < 0.01; TNF-α = *p* < 0.001). Significantly lower mRNA levels at 18 weeks were found for IL-17 (*p* < 0.01) and TNF-α (*p* < 0.05). Interferon-γ mRNA levels were variable during the monitored period, with it being higher (*p* < 0.001) in the DON group in the first week after birth, and then, contrastingly, it was lower *(p* < 0.01) in the same group three weeks after birth. No IFN-γ-related statistically significant differences (*p* > 0.05) were detected in the 18-week-old piglets (Figure 5). Our data show that intrauterine exposure to DON had a strong effect on the capacity of the blood lymphocytes to produce pro-inflammatory cytokines. The cytokine levels were generally lower in the DON group in comparison to the control group, and this effect remained even 18 weeks after birth.

## 3. Discussion

The present study focused on the impact of intrauterine DON exposure on piglets and their immune system. DON was administered to sows intravenously during the last three days of gestation. This artificial model allowed studying the impact of DON transferred from the sows’ blood to their offspring. The advantage of this model over the natural intake of DON via feed was that DON was not partially metabolized in the intestine to deepoxy-DON (DOM-1) [7,28]. The possible contribution of DON from the colostrum to the DON concentration in the piglets’ plasma was minimal. The DON concertation in the colostrum was very low and it was rapidly eliminated. It ranged from 0.56 to 0.78 ng/mL (less that the limit of quantification (LOQ) for colostrum; LOQ = 0.80 ng/mL, approximate concentrations) 4 h after delivery, identified in the mass spectrum, but the concentration was below the LOQ 8 h after delivery, and after 12 h was under limit of detection (LOD = 0.0.48 ng/mL for colostrum) [29]. A total DON dose of 300 µg was used to obtain a plasma concentration in the sows similar to that achieved after consuming contaminated feed. The available data show serum DON levels within the range of approximately 5–17 ng/mL after oral DON administration at a dose of 2.3 mg/kg [30].

Intrauterine transfer of DON was clearly documented in the present study, which is in agreement with earlier published data on the passage of DON through the placental barrier [22,23,24,25,26,27]. Our earlier published data show that DON was detectable in cord blood samples [29]. In the present study, the DON concentration in the plasma of the piglets was the highest at 12 h after delivery and decreased during the first week of life. However, DON persisted in the plasma of the piglets for up to 14 weeks after birth. This is in contrast to the data reported from older pigs, where DON was eliminated within 24 h after both intravenous (IV) and intragastric dosage [31]. Additionally, the authors of [32] reported DON elimination within hours after intravenous administration. As reported in our previous study, DON was undetectable in the plasma of the sows one week after IV dosage [29]. Whereas short-term DON metabolism in older pigs is also in accordance with data from adult human subjects [33], the long-term persistence of DON in the plasma of piglets was previously unreported, apart from one recent study (performed in pregnant sows consuming a diet naturally contaminated with DON). However, the data may be the result of the piglets eating contaminated sow feed in the late phase of the study, as discussed in [27]. This was not possible in our study, where DON was administered to sows intravenously, without any additional intentional dose postpartum. Furthermore, the feed for both sows and piglets was monitored and declared negative for DON (<LOD; LOD = 0.15 µg/kg for feed). It is highly probable that long-term DON persistence in the plasma of the piglets is a consequence of intrauterine exposure.

It is unclear why younger animals differ from older ones. Firstly, possible liver damage caused by DON exposure was tested. Biochemical analysis of the plasma ALT and AST levels did not show increased levels in comparison to the reference values. The reference values for determining increased levels of enzymes were 1.428 and 0.986 μkat/L for AST and ALT, respectively [19]. This means that the levels of both enzymes did not exceed the limits, implying that no liver damage had occurred. This was supported by the authors of [34], who reported the minimal effect of DON consumption on the liver biochemical parameters measured in the serum. Subsequently, the ability to eliminate DON in five-week-old piglets from the litter not exposed to DON was tested. Their capacity to metabolize DON was similar to that in piglets after intrauterine exposure. Based on these two observations, it can be concluded that the long-term persistence of DON seems to be a consequence of physiologically lower liver capacity in young piglets rather than a pathophysiological phenomenon caused by intrauterine exposure to DON.

Clarification of the long-term persistence of DON in the blood of the piglets could be found in the ontogeny of hepatic functions, specifically the pathways responsible for DON elimination. In pigs, only two DON metabolites have been described: glucuronide and deepoxy-DON (DOM-1) [35]. DOM-1 is a product of DON transformation by intestinal microbiota [28] and its contribution to DON elimination in pigs is weak [30,35]. Therefore, glucuronide formation seems to be the main pathway for DON elimination in pigs [36]. Glucuronide is a product of conjugation to glucuronic acid, and the DON-3-β-D-O-glucuronide type has been described in pigs [5]. Glucuronidation represents a major part of phase II metabolism and the reaction is catalyzed by enzyme uridine 5′-diphospho-glucuronosyltransferase (UGT). Several studies in different species have demonstrated a correlation of ontogeny with the presence or activity of UGT. In humans, it has been reported that several UGTs increase during postnatal ontogeny and genes for some are even absent in the fetal liver [37]. Similarly, low hepatic levels of UGT enzymes have described in fetuses and newborn sheep [38]. Recently, significant age-dependent hepatic activities of UGT were reported for pigs [39], with glucuronidation achieving peak activity at approximately 5–10 weeks after birth. The piglets after intrauterine DON exposure in our experiment were able to eliminate DON from their blood between 7 and 14 weeks. This suggests that the long-term persistence of DON in the plasma of the piglets could be a consequence of the physiologically lower activity of the hepatic enzymes involved in DON metabolism.

In addition, the possible impact of DON on the piglets’ immune system was studied. The effect of DON on the immune system parameters has been reported earlier in pigs [15,40]. In the present study, several significant differences between the piglets from the DON and the control groups were found in the immunological parameters such as plasma immunoglobulin level and T lymphocyte subset proportion, as well as in their capacity to produce cytokines. On the other hand, the monitored hematological parameters were not affected by intrauterine DON exposure, which is in accordance with other studies [41,42,43].

The epitheliochorial placenta in swine prevents the transfer of antibodies into the fetus during gestation. This means that immunoglobulin levels in newborn piglets are fully dependent on the antibodies acquired via colostrum. There were no significant differences in these levels between the DON and control groups in our study. In contrast, decreased IgG and IgA concentrations were reported in the serum of newborn piglets farrowed by sows fed an experimental diet containing DON from 89 ± 2 days of gestation [20]. This difference could be explained by a different time period in which the sows were exposed to DON (2–3 days versus 3 weeks) or by the influence of other mycotoxins in their feed (the diet contained 5.08 mg/kg of DON, 0.09 mg/kg of zearalenone, and 21.6 mg/kg of fusaric acid) [20]. However, our data show significantly lower levels of immunoglobulins at one week of life in the DON exposed group, followed by levels comparable with the control group at 3 and 18 weeks. Lower immunoglobulin levels as a consequence of DON exposure have been reported in mice [44,45]. In pigs, serum IgM and IgG levels have been shown to be significantly lower after feeding a diet containing DON for four weeks [43]. Another study described increased serum IgM and IgG levels after one dose of DON in the feed, but both IgM and IgG levels lacked significance when pigs were fed DON for four weeks [41]. It seems that data regarding serum Ig levels after DON exposure are rather inconsistent, and other factors could be involved.

Apart from serum immunoglobulin levels, the proportions of T cell subsets in the blood and the capacity to produce cytokines were monitored in the study. Both parameters were significantly affected by intrauterine exposure to DON. A significant impact of DON on T cells has previously been demonstrated in mice [46,47], cattle [48,49], poultry [49], and pigs [50,51,52]. In contrast, several studies of pigs have reported that long-term exposure to DON does not lead to any significant changes in the percentage of lymphocyte subpopulations [42,53]. Our present data show significant differences in percentages of all T cell subsets monitored—i.e., CD4+ Th cells, CD8hi Tc cells, γδ T cells, and T-regs—but only for the first and third weeks after birth. Noticeable postnatal development of T cell subsets occurred during the early postnatal period [54,55], suggesting that intrauterine exposure to DON has an impact on the development of T cells for a few weeks after birth. However, it does not affect the proportions of T cell subsets in older animals, as documented by the data from the 18-week-old piglets. The most decreased populations in the DON group were T-regs and CD8 positive CD4+ Th lymphocytes. The observed data are in accordance with those of Dąbrowski et al. [50], who reported a significant decrease in the porcine CD4+CD8+ subpopulation after per os DON administration across six weeks at a dose of 12 µg/kg body weight. For pigs, CD8α has been described as an activation marker of Th cells, thus double positive CD4+CD8+ cells present memory forms of Th lymphocytes [56]. The fact that DON has a significant impact on porcine T cell activation has also been described in an in vitro model [57]. Besides double positive CD4+CD8+ cells, T-regs were significantly decreased in the DON group at both early postnatal time points. T-regs play a crucial role in the maintenance of immune responses and T cell homeostasis [58]. In agreement with the confirmed role of T-regs in the regulation of both the adaptive and innate immune system of swine, their lower levels can significantly alter immune function during anti-infection response [59].

Finally, the capacity to produce T cell-related cytokines IFN-γ, IL-17, IL-2, and TNF-α after non-specific in vitro stimulation was monitored in the blood leukocytes from the DON-exposed and control piglets. The data show a statistically significant decrease of nearly all monitored cytokines in the piglets exposed to DON. This trend persisted even at 18 weeks after birth, when DON was not detectable in the plasma. As reported earlier, a significant decrease in the mRNA expression of IFN-γ, IL-17, IL-2, and TNF-α has been observed in vitro after a short-term (18 h) DON exposure [52]. The concentration of the DON in vitro culture was 1 and 10 ng/mL, which corresponds with that present in the plasma of the piglets after intrauterine exposure.

Taking together all of the data mentioned above, we can hypothesize that intrauterine DON exposure is a risk factor for the immune system of piglets. This is supported by data from other studies, where both antiviral and antibacterial responses were altered as a consequence of DON exposure. For example, the ingestion of a diet highly contaminated with DON greatly increased the effect of PRRSV (porcine reproductive and respiratory syndrome virus) infection on weight gain, lung lesions, and mortality [60]. During *Salmonella typhimurium* infection, DON amplified the inflammatory processes and increased the production of pro-inflammatory cytokines in the intestinal wall [61]. Besides the altered immune response against pathogens, a significant effect of DON on vaccination has also been observed [62,63]. Greater regulation on feed control for pregnant sows is essential for the prevention of the detrimental effect of DON on growing piglets. The use of mycotoxin detoxifiers with adsorbing or degrading properties are commonly used as feed additives for this purpose. Additionally, lactic bacteria from fermented cereals are good candidates for the reduction of mycotoxins in feed [64].

## 4. Conclusions

Maternal transfer results in the long-term persistence of DON in the blood of piglets, suggesting that young piglets do not have the capacity for DON metabolism. The presence of DON in the body changes the balance of the immune system, and, while the most significant impact was observed during the first three weeks of life, the decreased capacity to produce cytokines also remains in the later postnatal period. The presented data confirm the adverse effect of sows’ exposure to DON during pregnancy, showing transplacental transfer to the fetuses that passes into their blood in a toxic form as it cannot be metabolized, thereby causing significant harm to the litter.

## 5. Materials and Methods

### 5.1. Animals and Blood Sampling

Eight sows (Large White breed) and their litters (84 piglets in total) were included in the study. All sows were tested for DON prior to the experiment. DON (Sigma-Aldrich, St. Louis, MO, USA) was administered to four sows intravenously (IV) 2–3 days before delivery on a daily basis (300 µg of DON in 500 mL of infusion to provide plasma concentrations of 14.8–20.9 ng/mL) as reported previously [29]. Three sows were exposed to three doses (one dose per day) and one sow was exposed to two doses because earlier delivery occurred. An additional four sows were used as a negative control group. The study was carried out in two separate runs with four sows in each (two with DON and two controls). The piglets were suckling during the entire course of the experiment and were weaned at 28 days old. The feed for both sows and piglets was monitored and tested negative for DON (<LOD; LOD = 0.15 µg/kg for feed) throughout the study. Blood was collected from the jugular vein from all piglets 12 h after delivery and subsequently on a weekly basis at 1, 3, 7, 14, and 18 weeks of life. Blood samples were heparinized with 25 IU/mL of sodium heparin (Zentiva, Prague, Czech Republic). In the second experiment, six weaned piglets (35 days old) were added from the control group. DON was administered intraperitoneally (IP) at one dose of 30 µg of DON in 5 mL of infusion. Heparinized blood samples were collected before IP DON administration, and then at 12, 24, 48, and 72 h after IP DON administration and then once a week for 20 weeks.

The animal care and use protocols were approved by the Ethical Committee of the Veterinary Research Institute according to guidelines in the Animal Protection Act and were subsequently approved by the Branch Commission for Animal Welfare of the Ministry of Agriculture of the Czech Republic (reference number 45933/2016–MZE–17214, Date: 16 August 2016).

### 5.2. Liquid Chromatography in Combination with High-Resolution Mass Spectrometry (LC–MS/MS (HR))

Plasma concentrations of DON were analyzed using liquid chromatography combined with a high resolution Orbitrap-based mass spectrometer (LC–MS/MS (HR)). The method was validated for DON in porcine plasma according to EU regulation 657/2002/EC and published previously [29]. Briefly, pre-treatment of the samples involved a deproteinization step with methanol, followed by a purification step by solid phase extraction (HLB cartridges). Chromatographic separation was performed on a C18 Luna Omega column with a particle size of 1.6 µm (Phenomenex, Torrance, CA, USA) using a water–methanol mobile phase. Detection of analytes was achieved on the tandem hybrid mass spectrometer Q Exactive (Thermo Fisher Scientific, Waltham, MA, USA), with a heated electrospray ionization probe measured in positive mode (H–ESI+). For confirmation of the presence of these analytes in the porcine plasma, matching of the retention time with mass accuracy for the precursor ion from MS and product ions from MS/MS was used. A deuterium isotopically labeled internal standard 3-Acetyl-d_3_-deoxynivalenol (Sigma-Aldrich, St. Louis, MO, USA) and a matrix-matched calibration curve were employed for quantification. The performance of the developed method was verified by validation; the linear range of quantification was 0.5–20 Lg/L and the correlation coefficient (R^2^) was >0.999 for all calibration curves. The limit of detection (LOD) for DON in the plasma was 0.24 ng/mL and the limit of quantification (LOQ) was 0.39 ng/mL. The relative standard deviation values were less than 9.5% and the recovery method was in the range of 98–105% in the plasma [29]. Additionally, feed was controlled for DON with an LOD of 0.15 µg/kg.

### 5.3. Aspartate Aminotransferase (AST) and Alanine Aminotransferase (ALT) Determination in the Plasma

Enzymes alanine aminotransferase (ALT) and aspartate aminotransferase (AST) were measured in the blood plasma collected from 3-week-old piglets. Values were determined by the enzymatic–colorimetric method using the automated chemical analyzer BS-200 (Mindray, Shenzhen, China) and commercial kits (Greiner Diagnostic GmbH, Bern, Switzerland) according to the manufacturer’s protocol.

### 5.4. Hematological Parameters

Red blood cells (RBCs), nucleated red blood cells (NRBCs), hematocrit (HTK), hemoglobin (Hb), and total white blood cell counts (WBCs) were assessed using the auto hematology analyzer BC-2800Vet (Mindray, Shenzhen, China). Differential leukocyte counts were determined by counting 200 cells in the blood smears stained with May–Grünwald and Giemsa–Romanowski (Penta, Prague, Czech Republic).

### 5.5. Serum Immunoglobulin Concentration

The total serum immunoglobulin (Ig) concentration was determined spectrophotometrically by measuring the turbidity resulting from the addition of zinc sulfate to the serum. The procedure was a modification of the method described by McEwan et al. [65]. Twenty-five microliters of serum were mixed with 1.3 mL of a 0.7 mM solution of zinc sulfate, of pH 5.8, and the resulting turbidity was measured at 590 nm after 1-h incubation with gentle shaking at room temperature. A blank (serum with phosphate-buffered saline) was run with each serum sample. The concentration of total Ig in the tested sera was calculated from the calibration curves of the standards created using various Ig concentrations.

### 5.6. Flow Cytometry Analysis of the Lymphocyte Subpopulations in the Blood

The lymphocyte subpopulations, including T-regs, were analyzed using flow cytometry and protocols for T cell surface marker staining and intracellular transcription factor Forkhead-box p3 (Foxp3) staining. First, erythrocytes were removed from the heparinized blood samples using an ammonium chloride solution, and then single-cell suspensions of leukocytes were stained as described previously [66]. A six-color panel CD3/CD4/CD8/γδTCR/propidium iodide was used for T cell subpopulation identification. The primary mouse anti-pig antibodies were as follows: CD3ε (PerCP-Cy™5.5-conjugated, BB23-8E6-8C8, BD Biosciences, Franklin Lakes, NY, USA), γδTCR (unconjugated, PGBL22A, IgG1, WSU, Pullman, Wash., USA), CD4 (unconjugated, 10.2H2, IgG2b, WSU, Pullman, WA, USA), and CD8α (unconjugated, 76-2-11, IgG2a, WSU). As secondary antibodies, isotype-specific fluorochrome-labeled goat anti-mouse antibodies were used: Alexa Fluor 488 (IgG2a, Invitrogen—Thermo Fisher Scientific), Alexa Fluor 647 (IgG2b, Invitrogen—Thermo Fisher Scientific), and Phycoerythrin (IgG1, Invitrogen—Thermo Fisher Scientific). Regulatory T cells were characterized as FoxP3—positive CD4+CD25hi cells. First, samples were labeled with monoclonal primary antibodies against CD4 (10.2H2, IgG2b, WSU, Pullman, Wash., USA) and CD25 (unconjugated, PGBL25A, IgG1, WSU). Subsequently, the samples were labeled with secondary goat anti-mouse antibodies Alexa Fluor 488 (IgG1, Invitrogen—Thermo Fisher Scientific), APC-Cy7 (IgG2b, Southern Biotech, Birmingham, Ala., USA), and a probe for dead cell identification (LIVE/DEAD™ Fixable Yellow Dead Cell Stain Kit, for 405 nm excitation; Invitrogen—Thermo Fisher Scientific). Finally, samples were fixed and permeabilized using the eBioscience Foxp3/Transcription Factor Staining Buffer Set (Invitrogen—Thermo Fisher Scientific) according to the manufacturer’s instructions and labeled with anti-Foxp3 antibody (Phycoerythrin-conjugated, FJK-16s, eBioscience—Thermo Fisher Scientific).

**Figure A1:**
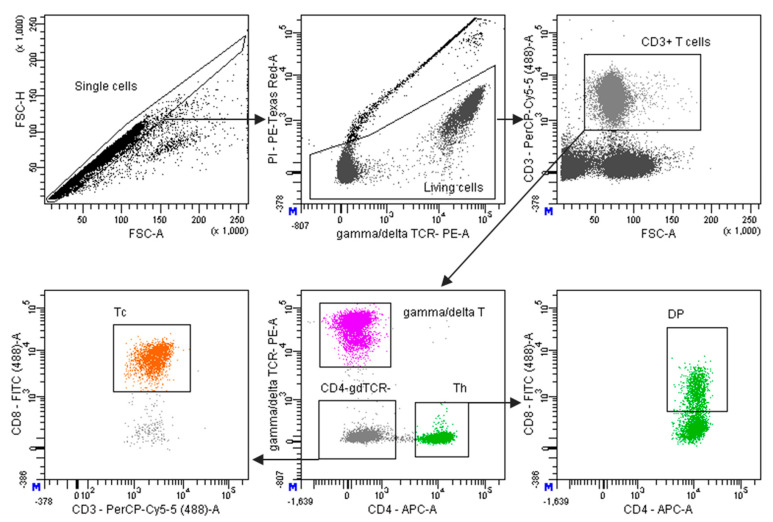
Gating strategy—flow cytometry analysis of the T lymphocyte subpopulations. Doublets and dead cells identified by propidium iodide (PI) staining were excluded. Subsequently, the T cell subpopulations were defined from all CD3+ cells: γδ T cells (CD3+γδTCR+CD4-CD8+/-), cytotoxic T cells (Tc; CD3+γδTCR-CD4-CD8+), helper T cells (Th; CD3+γδTCR-CD4+CD8+/-), and double positive CD4+CD8+ cells (DP; CD3+γδTCR-CD4+CD8+). The percentage of population positive for each population was evaluated. Dot plots show data from one representative sample. Values for the Tc cells were calculated as a percentage of all CD3+ T cells.

**Figure A2:**
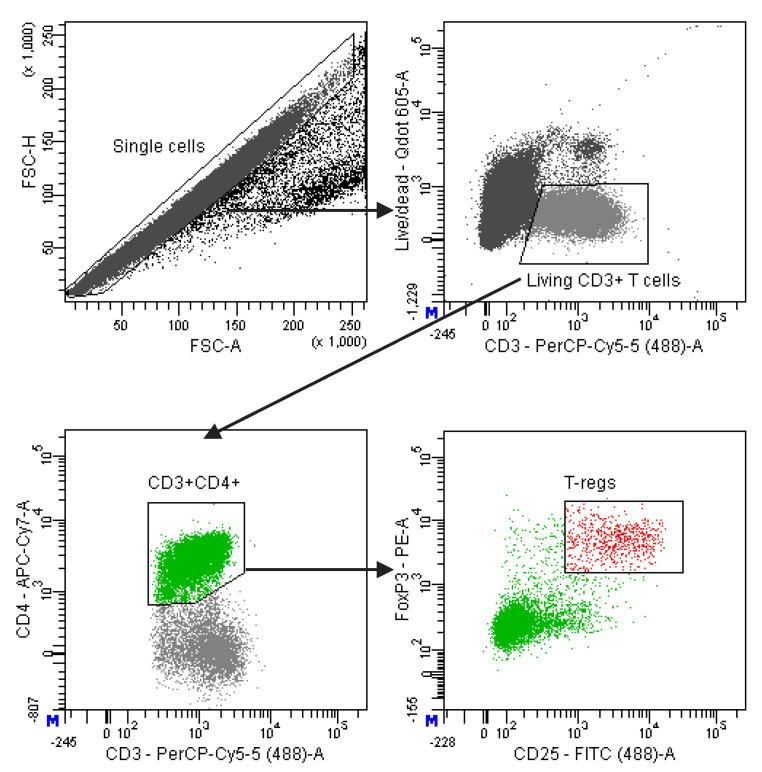
Gating strategy—flow cytometry analysis of T-regs. Doublets and dead cells identified by the live/dead probe (Fixable Yellow Dead Cell Stain Kit for 405-nm excitation) were excluded. Subsequently, T-regs were defined from all CD3+CD4+ cells as the CD3+CD4+CD25+FoxP3+ population. Dot plots show data from one representative sample.

The stained samples were acquired on a flow cytometer BD FACSAria Fusion operated using Diva software (BD). Doublets and dead cells were excluded from the analysis using Diva software, and T cell subpopulations were analyzed according to Figure A1 and Figure A2.

### 5.7. Non-Specific Stimulation of Blood Leukocytes and Cytokine Gene Expression Determination by Quantitative RT-PCR Analysis

Whole heparinized blood was diluted at a 1:1 ratio with RPMI-1640 medium (Sigma-Aldrich) supplemented with antibiotics (100 IU/mL penicillin and 100 μg/mL streptomycin; PAA). Samples were stimulated with 15 nM/mL phorbol myristate acetate (PMA; Sigma-Aldrich) and 1 μg/mL ionomycin (Sigma-Aldrich) in a 96-well plate for 5 h. Afterward, erythrocytes were lysed with a hemolytic ammonium chloride solution with two subsequent washing steps using phosphate-buffered saline. After the final wash, samples were centrifuged and pellets were resuspended in Tri-Reagent RT (Molecular Research Center, Cincinnati, OH, USA) and stored at −80 °C until RNA isolation.

The methods of RNA isolation, reverse transcription, and qRT-PCR have been described previously [52]. Briefly, the RNA phase was isolated from the Tri-Reagent RT homogenate mixed with bromoanisole by separation in a refrigerated centrifuge with subsequent RNA purification using an RNeasy Mini Kit (Qiagen, Hilden, Germany). The RNA was then reverse transcribed with M-MLV reverse transcriptase (Invitrogen—Thermo Fisher Scientific, Waltham, Mass., USA) using oligo-dT primers. Measurements were performed using the QuantiTect SYBR Green PCR Kit (Qiagen), LightCycler 480 II with a 384-well plate block (Roche, Basel, Switzerland), and Innovadyne Nanodrop robot (IDEX Health & Science LLC, Oak Harbor, WA, USA). Hypoxanthine phosphoribosyltransferase (HPRT) mRNA was used as a housekeeping reference gene. The threshold cycle values (*C*_t_) of the gene of interest were first normalized to the *C*_t_ value of the HPRT reference mRNA (Δ*C*_t_), and then the normalized mRNA levels were calculated as 2^(−ΔC^_t_^)^. The measured cytokines included interferon (IFN)-γ, interleukin (IL)-17, IL-2, and tumor necrosis factor (TNF)-α. The normalized mRNA levels of the cytokines are shown as “HPRT units”. Primers used in the study were adopted from Volf et al. [67] (HPRT and TNF-α), Stepanova et al. [68] (IFN-γ), and Stepanova et al. [66] (IL-17). The primer for IL-2 was designed in the study using Primer3 Software (forward 5′–3′ GGAATCAATGAACAATATCAACG, reverse 5′–3′ TTTGTTCAGAAATTCAACAGCA).

### 5.8. Statistical Analysis

The data were analyzed using non-parametric Mann–Whitney tests. *p*-values < 0.05 were considered significant (* *p* < 0.05, ** *p* < 0.01, and *** *p* < 0.001). Outliers were excluded from the analysis according to the Grubbs’ test. Correlation age versus DON concentration was analyzed using Spearman’s rank correlation tests. All calculations and graphs in Figure 3, Figure 4 and Figure 5 were performed with GraphPad prism software (GraphPad Software, San Diego, CA, USA). Figure 1 and Figure 2 were created using Minitab software (Minitab, Ltd., Coventry, UK).

## Figures and Tables

**Table 1:** The hematological parameters determined in the piglets after intrauterine exposure to DON. Data are expressed as mean (standard deviation). None of the parameters show statistically significant differences between the control and DON groups (Mann–Whitney test; *p* ˃ 0.05).

	1 Week		3 Weeks		18 Weeks	
	Control (*n* = 36)	DON (*n* = 39)	Control (*n* = 33)	DON (*n* = 38)	Control (*n* = 27)	DON (*n* = 32)
RBCs (1012/L)	4.1 (0.9)	3.9 (0.7)	5.4 (0.5)	5.4 (0.5)	6.8 (0.4)	6.9 (0.4)
HTK (%)	27.9 (4.5)	28.1 (3.1)	30.8 (4.0)	30.9 (4.2)	35.3 (2.0)	38.1 (2.6)
Hb (g/L)	87.3 (15.8)	88.5 (10.8)	101.1 (13.0)	102.4 (15.2)	113.9 (6.8)	121.8 (9.2)
WBC (109/L)	13.9 (10.0)	11.7 (3.8)	8.3 (3.0)	7.5 (2.4)	14.5 (3.0)	12.8 (2.9)
Lymphocytes (%)	47.5 (18.1)	53.2 (16.2)	59.9 (14.9)	62.0 (13.7)	76.0 (9.5)	80.5 (9.6)
Neutrophils (%)	49.4 (18.5)	43.4 (16.5)	38.1 (14.4)	36.9 (13.6)	21.2 (9.7)	17.0 (9.4)
Band neutrophils (%)	1.4 (1.4)	1.7 (1.9)	0.9 (0.7)	0.4 (0.5)	0.3 (0.6)	0.4 (0.5)
Monocytes (%)	0.5 (0.7)	0.7 (0.7)	0.3 (0.5)	0.2 (0.3)	0.6 (0.7)	0.5 (0.8)
Eosinophils (%)	0.7 (0.5)	0.5 (0.6)	0.5 (0.5)	0.3 (0.4)	1.1. (0.8)	1.1 (0.8)
Basophils (%)	0.4 (0.5)	0.5 (0.6)	0.3 (0.4)	0.2 (0.3)	0.9 (0.9)	0.6 (0.6)
NRBCs (counts/100 WBCs)	14.1 (20.6)	12.9 (12.0)	5.4 (5.5)	3.0 (3.8)	1.1 (1.3)	0.6 (0.7)

RBC, red blood cells; HTK, hematocrit; Hb, hemoglobin; WBC, total white blood cell count; NRBC, nucleated red blood cells.
